# Nanoscale Artificial Plasmonic Lattice in Self‐Assembled Vertically Aligned Nitride–Metal Hybrid Metamaterials

**DOI:** 10.1002/advs.201800416

**Published:** 2018-04-27

**Authors:** Jijie Huang, Xuejing Wang, Nicki L. Hogan, Shengxiang Wu, Ping Lu, Zhe Fan, Yaomin Dai, Beibei Zeng, Ryan Starko‐Bowes, Jie Jian, Han Wang, Leigang Li, Rohit P. Prasankumar, Dmitry Yarotski, Matthew Sheldon, Hou‐Tong Chen, Zubin Jacob, Xinghang Zhang, Haiyan Wang

**Affiliations:** ^1^ School of Material Engineering Purdue University West Lafayette IN 47907‐2045 USA; ^2^ Department of Chemistry Texas A&M University College Station TX 77840 USA; ^3^ Sandia National Laboratories Albuquerque NM 87185 USA; ^4^ Los Alamos National Laboratory Los Alamos NM 87545 USA; ^5^ School of Electrical and Computer Engineering Purdue University West Lafayette IN 47906 USA

**Keywords:** artificial plasmonic lattice, metal–nitride nanocomposites, metamaterials, plasmonics, self‐assembly, vertically aligned nanocomposite

## Abstract

Nanoscale metamaterials exhibit extraordinary optical properties and are proposed for various technological applications. Here, a new class of novel nanoscale two‐phase hybrid metamaterials is achieved by combining two major classes of traditional plasmonic materials, metals (e.g., Au) and transition metal nitrides (e.g., TaN, TiN, and ZrN) in an epitaxial thin film form via the vertically aligned nanocomposite platform. By properly controlling the nucleation of the two phases, the nanoscale artificial plasmonic lattices (APLs) consisting of highly ordered hexagonal close packed Au nanopillars in a TaN matrix are demonstrated. More specifically, uniform Au nanopillars with an average diameter of 3 nm are embedded in epitaxial TaN platform and thus form highly 3D ordered APL nanoscale metamaterials. Novel optical properties include highly anisotropic reflectance, obvious nonlinear optical properties indicating inversion symmetry breaking of the hybrid material, large permittivity tuning and negative permittivity response over a broad wavelength regime, and superior mechanical strength and ductility. The study demonstrates the novelty of the new hybrid plasmonic scheme with great potentials in versatile material selection, and, tunable APL spacing and pillar dimension, all important steps toward future designable hybrid plasmonic materials.

Designed metamaterials, such as metainterfaces with subwavelength size, exhibit unique optical properties and open up a new platform toward various applications. Biosensing,^1^, ^2^ superlenses,^3^, ^4^ subwavelength optics,^5^, ^6^, ^7^ as well as cloaking,^8^, ^9^, ^10^ have been achieved, mostly by artificially designed metallic metamaterials (especially Au, Ag), due to their extraordinary optical properties, such as strong plasmon‐mediated energy confinement, negative refraction, surface plasmon propagation, and zero scattering. More importantly, it has been reported that closely spaced metal nanoparticles show interesting properties such as tunable optical response in the visible range,^11^ 3D photonic crystals with robust photonic bandgaps,^12^ and theoretical prediction of plasmonics edge states in metallic honeycomb‐like lattices.^13^ Furthermore, artificially designed metasurfaces, which consist of close packed subwavelength resonators, can modify the scattered wave front at the deep subwavelength scale.^14^

Most of these metallic metamaterials were fabricated by electrochemical deposition into a porous alumina template,^1^, ^15^ combined focused ion beam and electroplating,^16^, ^17^ electron beam evaporation,^18^, ^19^ cascaded sheet admittance (patterned metallic sheets),^20^, ^21^ as well as electron beam lithography.^22^, ^23^ Some extraordinary optical properties in the visible range have been investigated for these hyperbolic metamaterials,^15^, ^24^, ^25^ such as the silver (Ag)^15^ and gold (Au)^24^, ^25^ nanowires in porous alumina template. The limitations lie in the costly template, the complicated and tedious nanofabrication methods, and the challenges for large‐scale processing with nanoscale features. Alternatively, simple self‐assembly approaches could provide a more practical route for large‐scale processing of these designed nanostructured metamaterials.^26^, ^27^ For example, 3D tungsten photonic crystal was deposited using a flow‐tube atomic layer deposition, which exhibited excellent thermal stability for effective solar thermo‐photovoltaic devices.^27^

In this work, a new hybrid metamaterial concept is proposed to construct highly ordered nanoscale plasmonics lattice structures using two‐phase hybrid metamaterials, as illustrated in **Figure** **1**a. The vertical nanopillars form an ordered hexagonal close‐packed artificial plasmonic lattice (APL) structure by a direct self‐assembled epitaxial thin film growth in the vertically aligned nanocomposite (VAN) form, as shown in the plan‐view transmission electron microscopy (TEM) image in Figure 1b and the corresponding schematic illustration in Figure 1c. More specifically, the metal–nitride hybrid metamaterial systems are chosen for this demonstration. Both Au and nitrides have been demonstrated as effective plasmonic materials. Au is chosen as the metallic nanopillars as it has been widely demonstrated as various plasmonic structures and surface‐enhanced Raman scattering.^28^, ^29^, ^30^ The matrix material is from the family of transition metal nitrides, including titanium nitride (TiN), tantalum nitride (TaN), zirconium nitride (ZrN), and hafnium nitride (HfN). They have been considered as one of the alternative candidates beyond metallic plasmonic materials to overcome some of the drawbacks such as interband transition loss and high temperature incompatibility.^31^, ^32^ Their plasmonic performance is particularly in the visible and near‐infrared regimes.^33^ Nitrides could be complementary to the primary metallic lattice in the proposed hybrid plasmonic systems.

**Figure 1:**
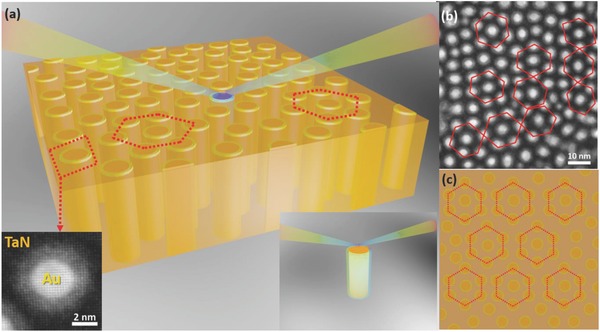
a) Schematic illustration of the designed TaN–Au metamaterial with ordered honeycomb‐like Au nanopillar lattice. b) Low‐magnification plan‐view TEM image presents the hexagonal lattice in a large scale. c) Top view of the schematic illustration.

The VAN thin film platform provides a unique and highly anisotropic nanostructure with vertically aligned nanopillars in matrix. Most of the VAN structures have been demonstrated in oxide–oxide systems,^34^, ^35^, ^36^, ^37^, ^38^ because of the excellent growth compatibility between the oxide–oxide systems. The growth of nitride–metal plasmonic lattice structures posed significant difficulties due to the large difference in growth kinetics of the two phases, i.e., a metal and a nitride in this case. On the other hand, the main advantages of the Au–nitride hybrid systems are: 1) the reasonable lattice matching between the Au pillars (*a*_Au_ = 4.065 Å), TaN (*a*_TaN_ = 4.37 Å), and TiN (*a*_TiN_ = 4.249 Å), all in the face centered cubic structure, allows the high quality epitaxial growth of the proposed APL structure; 2) the different surface energy favors the formation of Au pillars (a high surface energy leads to a 3D pillar growth) in the nitride matrix (a low surface energy leads to a 2D matrix growth) which enables the proposed plasmonic lattice structure. In this work, several nitride matrices have been explored and tested to form such two‐phase hybrid metamaterials and confirmed by the plan‐view and cross‐section TEM/scanning transmission electron microscopy (STEM) analysis. The formation of such highly ordered hexagonal Au‐nanopillar plasmonic lattice and its tunability are further discussed and correlated with their unique optical properties such as exceptional plasmonic response. The metal–nitride hybrid metamaterials with the APL present enormous opportunities in novel hybrid plasmonic metasurface designs with a wide range of material selections, tunable wavelength ranges, and great potentials in scalability.

X‐ray diffraction (XRD) characterization has been applied to determine the crystallinity of the Au–nitrides, as shown in Figures S1–S3 with related discussions (Supporting Information). To explore the actual 3D morphology of the nitride–metal hybrid film on different substrates, both plan‐view and cross‐sectional TEM studies have been carried out. The overview 3D morphology of the TaN–Au on MgO can be seen in the TEM illustration in **Figure** **2**a. It is clear that vertically aligned Au nanopillars are grown highly epitaxially and uniformly embedded into the epitaxial TaN matrix. Plan‐view STEM image (in high angle annular dark field (HAADF) mode) in Figure 2b shows very interesting hexagonal close packed Au nanopillars in TaN matrix as the proposed artificial hexagonal lattice in the matrix. This unique structure is believed to be caused by the strain compensation of TaN–MgO and Au–MgO. More specifically, TaN has a lattice parameter of 4.37 Å and a 3.68% compressive strain with MgO (*a*_MgO_ = 4.212 Å) while Au has a lattice parameter of 4.065 Å and a 3.55% tensile strain with MgO. Such difference in the strain state of the two‐phase results in the well‐distributed Au pillar in TaN matrix to make the overall mismatch strain on the MgO substrate minimal. However in the case of TiN–Au, such strain compensation effect is minimal as TiN (*a*_TiN_ = 4.231 Å) has almost perfect lattice matching with MgO (*a*_MgO_ = 4.212 Å). Thus, the Au nanopillars are uniformly distributed in TiN matrix but not with the perfect ordering as the case for TaN–Au. The theoretical in‐plane and out‐of‐plane lattice misfit, dislocation spacing between the materials and substrates are shown in **Table** **1**. These values were calculated based on bulk lattice parameters at room temperature. The ordered hybrid nanostructure is therefore named as APL. The circular Au nanopillars are surrounded by a uniform cloud‐like interfacial area. The STEM images under the HAADF mode show the image contrast proportional to ∼*Z* ^2^. Thus, Au shows the brightest contrast, TaN is the darkest and the interface area is in intermediate contrast. This suggests that the interfacial area contains either more Ta or less nitrogen comparing to the matrix area, or potentially some Au interdiffusion. This is later confirmed as stoichiometric TaN in the interface area and Ta_3_N_5_ in the matrix area based on the optical property analysis in the following section. Plan‐view energy‐dispersive X‐ray spectroscopy (EDS) elemental mapping (Figure 2c) was further conducted to confirm the elemental distribution in‐plane. The Au‐ and Ta‐elemental mappings clearly indicate the sharp interface between the Au nanopillars and TaN matrix without any obvious Au interdiffusion. This further confirms the possibility of nitrogen‐deficient or Ta‐rich interface region comparing to the matrix region, possibly due to the high interfacial strain at the interface areas. High resolution STEM HAADF as well as its corresponding inverse Fourier‐filtered image were conducted to explore the detailed microstructure of the nanopillars, as shown in Figure 2d,e, respectively. The misfit discontinuous stripes present dislocations or stacking faults, which has been presented by “” in Figure 2e. The epitaxial circular Au nanopillars have a uniform diameter of ≈3 nm and the width of the strained annulus region is ≈1.3 nm. The average distance is ≈10 nm from one pillar center to another and ≈7 nm from edge to edge (note that the nanopillar geometry can be further tuned by controlling the growth condition, such as laser frequency and deposition temperatures). From the masked inverse fast Fourier transform (FFT) image, very few dislocations can be identified in the interface area and thus a high lattice strain of 7.23% can be maintained in the interface areas which might be account for the formation of the highly strained N‐deficient or Ta‐rich interface regions. The lattices are extended nicely from the matrix to the nanopillars with a clear cube‐in‐cube relationship between the two phases, which again confirms the excellent epitaxial quality of the overall hybrid APL system. A low‐mag STEM image is shown in Figure S4 (Supporting Information) to demonstrate that the Au nanopillars are uniformly distributed in the entire film.

**Figure 2:**
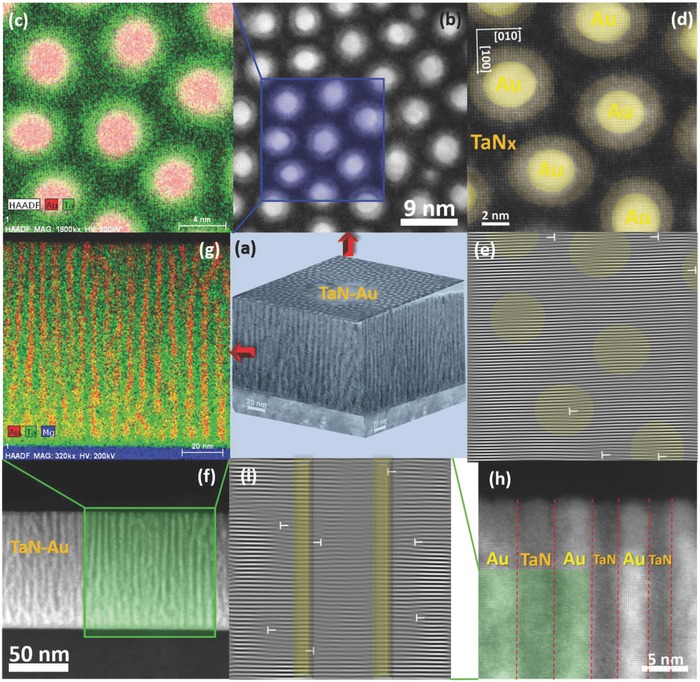
3D microstructural characteristics of TaN–Au thin film on MgO substrate. a) 3D‐viewed diagram of TaN–Au thin film generated by plan‐view and cross‐sectional transmission electron microscopy (TEM) images. b) Typically plan‐view STEM image of the epitaxial TaN–Au film with c) corresponding EDS mapping of selected area. d) High‐resolution plan‐view STEM HAADF image of the film with e) its corresponding masked inverse FFT image. f) Typically cross‐sectional STEM image of the film with g) corresponding EDS mapping of selected area. h) High resolution cross‐sectional STEM HAADF image of the film with i) its corresponding masked inverse FFT image.

**Table 1:** The list of theoretical in‐plane (IP) and out‐of‐plane (OP) lattice misfit, the dislocation spacing between the materials and the substrates in this work, calculated by the bulk lattice parameters at room temperature

	Misfit ratio: 2(*a*_s_ − *a*_f_)/(*a*_s_ + *a*_f_) × 100%	Estimated misfit dislocation spacing: *d*_002s_ × *d*_002f_/|*d*_002s_ − *d*_002f_| [nm]
TiN/MgO (IP)	−0.9	4.86
Au/MgO (IP)	3.55	5.82
TaN/MgO (IP)	−3.68	5.82
Au/STO (IP)	−4.02	4.96
TiN/STO (IP)	−8.44	2.41
TaN/STO (IP)	−11.24	1.83
TiN/Au (OP)	4.43	4.69
TaN/Au (OP)	7.23	2.91

Cross‐sectional STEM and corresponding EDS mapping of selected area were conducted to fully investigate the 3D morphology of the films, as shown in Figure 2f,g, respectively. Highly ordered vertically aligned Au nanopillars can be observed with almost the same pillar‐to‐pillar spacing, and they are grown straight up through the entire thickness of the film. By enlarging a representative area in Figure 2h, a perfect lattice matching relationship of TaN (001)//Au (001) and TaN (100)//Au (100) can be determined. Some dislocations and induced strain can be identified in the interface area, revealed by inverse Fourier‐filtered image in Figure 2i, which is consistent with the plan‐view results above. However, the TaN–Au grown on SrTiO_3_ (STO) substrates exhibits different microstructure, possibly due to the larger lattice mismatch between STO (*a*_STO_ = 3.90 Å) and TaN (*a*_TaN_ = 4.37 Å), as well as the different growth modes. The Au nanopillars are not as straight as the one on MgO substrate, as shown in Figure S5 (Supporting Information).

The formation of such highly ordered self‐assembled nitride–metal nanocomposite as APL hybrid metamaterials is quite interesting and worth further investigation. Compared to the extensively studied oxide–oxide VAN^34^, ^35^, ^36^, ^37^, ^38^ and recently reported metal–oxide VAN,^26^, ^39^ the nitride–metal VAN exhibits surprisingly ordered Au nanopillars, forming the APL hybrid systems. The growth of the APL hybrid structure is dominated by the different growth mechanisms of the metal and nitride in the systems. First, Au and TaN are immiscible in the film, as confirmed by the microstructure study. Second is the nucleation of the different phases with different surface energy. The surface energy of Au (001), TaN (001), and MgO (001) are 1.627 J m^−2^,^40^ ≈1 J m^−2^,^41^ and ≈1.15 J m^−2^,^42^ respectively. Therefore, Au adatoms diffuse and nucleate as 3D islands and thus lead to the growth of nanopillars (the Volmer–Weber 3D island growth mode), due to the higher interfacial energy γ_AM_ (lower wettability) with MgO substrate. On the other hand, because of the lower interfacial energy γ_TM_ (higher wettability) between TaN and MgO, TaN follows the 2D Frank–van der Merve mode or 2D+3D Stranski–Krastanov mode, which leads to the layer‐by‐layer growth and forms the planar matrix. More important is the exciting hexagonal closely packed APL structure consisted of highly ordered Au nanopillars. The pillar spacing is around 10 nm which is related to the lattice mismatch between the film and the substrate, which is currently under investigation.

Insight into the morphology and the plasmonic behavior of the APL hybrid structures is also provided by analysis of the Raman scattering signal, as summarized in **Figure** **3**a,b. These data are plotted with the peak scattering intensity normalized in order to emphasize the comparison of spectral features between the APL hybrid structures and the corresponding pure nitride control samples. It is noted that all APL structures showed large spectral bands of increased Raman signal, between 100% and 700% enhancement, in comparison with the control pure nitride samples that did not contain Au nanopillars. Un‐normalized Raman spectra, comparing the absolute magnitude of the Raman signal from APL hybrid structures and control substrates are provided in Figure S6 (Supporting Information). The plasmonic enhancement of Raman scattering provided by nanostructured Au metal is well known, and the enhanced Raman signal observed here demonstrates the high optical quality of the Au nanopillars in the APL structure.

**Figure 3:**
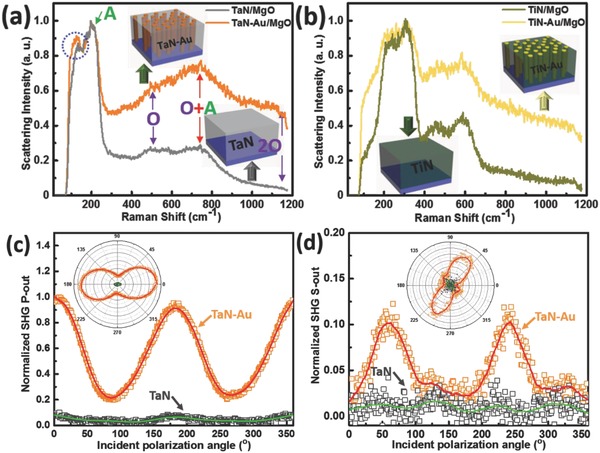
Normalized Raman spectra of a) TaN and TaN–Au on MgO, and b) TiN and TiN–Au on MgO; The insets are their corresponding schematic illustrations. Normalized SHG intensity as a function of incident polarization angle with output polarization fixed at c) 0° (P‐out) and d) 90° (S‐out). The insets are their corresponding polar plots of measured SHG intensity versus incident polarization angle.

Because the plasmonic field enhancement decays quickly in the vicinity around the metal, as indicated in the optical field profiles in Figure 3a,b and Figure S7a,b (Supporting Information), the enhanced Raman signal primarily provides structural information about the interface region between the metal nanopillar and the nitride matrix, allowing a detailed comparison of the nitride located at this interface region versus the nitride that comprises the bulk of the control samples. More specifically, when comparing the Raman data (Figure 3a), two primary features have been observed corresponding to the first‐order acoustic mode (A) centered near 200 cm^−1^ as well as a broad first‐order optical mode (O) at 550 cm^−1^ for TaN, and at similar shifts for the other metal nitrides. Other spectral features correspond to linear combinations of these scattering peaks. The presence of these first‐order modes in all of the scattering spectra indicates that the metal nitride has adopted some non‐centrosymmetric lattice parameters, since pure crystalline phases of TaN, and TiN are centrosymmetric and typically exhibit no Raman activity for first‐order modes. The occurrence of these spectral features in TaN is usually interpreted as indication of modified stoichiometry,^43^ either excess Ta or excess N. Furthermore, the decrease in the relative ratio of the A and O modes between the control sample and the APL samples indicates that the nitride signal is more stoichiometric in the APL structures.^43^ Given that the enhanced Raman signal from the APL substrates is indicative of the nitride located at the interface region between the Au pillar and the bulk matrix, this trend, taken together with the HAADF analysis above, suggests that the interfacial region directly surrounding the Au pillars is more stoichiometric TaN, whereas there is a greater excess of nitrogen in the nitride matrix (TaN*_X_*, *X* > 1, e.g., a mixture of TaN and Ta_3_N_5_). Furthermore, in addition to this stoichiometric information, a slight downshift of Raman peaks (≈10 cm^−1^) from the APL films is observed. The shift is highlighted in the dashed blue circle in Figure 3a. This frequency shift can be attributed to compressive lattice strain at interface of the Au pillar and the nitride matrix,^44^ which is consistent with the lattice mismatch between the two materials. The non‐centrosymmetry of TaN in TaN–Au APL nanostructures can be further confirmed by second harmonic generation (SHG) measurement. The comparison of TaN and TaN–Au for SHG intensity as a function of incident polarization angle with output polarization fixed at 0° (P‐out) and 90° (S‐out) is shown in Figure 3c,d, respectively. The insets are their corresponding polar plots of measured SHG intensity versus incident polarization angle. The input polarization was scanned at an angle resolution of 1° (0.5° for the half‐wave plate), and the angle of incident was set at 45°. Apparently, pure TaN presents almost negligible SHG signal, due to the inversion symmetric structure of TaN. However, interestingly, TaN–Au exhibits much stronger SHG response compared to pure TaN. Several factors can be considered for this enhancement, including TaN–Au interface, as well as the broken inversion symmetry of TaN lattices induced by the high density TaN/Au interfaces, all consistent with the above microstructure and Raman characterizations.

A crucial design element in the APL hybrid structures is the tunable optical properties afforded by the high level of morphological control during growth. Opportunities for engineering novel optical behavior depend on robust plasmon resonances from each nanopillar, and the electromagnetic coupling between nanopillars based on the dielectric properties of the matrix materials as well as the local and mesoscale ordering at spatial frequencies that couple to propagating radiation. It is noted that, until this report, large‐area plasmonic structures that provide nanometer‐sized Au nanopillars separated by less than 10 nm have not been demonstrated, and thus this platform may open new pathways for a variety of functional optical metamaterials. The main proposed advantage of hyperbolic metamaterials, corresponding to the structures fabricated in this study, is that the greatly increased density of states can enhance nonlinear optical phenomenon, such as the SHG demonstrated here, as well as exotic optical effects such as superlensing and superfocusing that result from a negative index of refraction. A higher pillar density and smaller pillar radii mean that the metamaterial can achieve these effects over a larger spectral range, as this unique behavior requires that composite structural features are small compared with the wavelength of light. An initial characterization of the optical response of the APL hybrid structures is performed to highlight the role of the plasmonic response of the nanopillars. The overall sample reflectivity was measured at normal incidence and compared with full‐wave electromagnetic simulations of the sample geometry. It is worth noting that full‐wave electromagnetic simulations are adopted here to account for the magnitude and spectral dispersion accurately, as these parameters are very sensitive to features of the interface of the metal pillars and the degree of ordering. Note, the transmittance of TaN, TaN–Au, TiN, and TiN–Au is almost zero throughout the visible wavelength regime, as shown in Figure S8a,b (Supporting Information). Structural features in the simulations are based on the STEM and high‐resolution TEM (HRTEM) analysis. Further details of the simulation are provided in the Supporting Information. The optical data are summarized in **Figure** **4** for the Au–TaN APL. In comparison with a control sample of pure TaN thin film, the measured reflection spectra exhibit generally increased reflectance over the entire spectral range, except near a broad feature centered around 475 nm. This decrease in reflectance corresponds to enhanced absorption due to the plasmon resonance of individual Au nanopillars. As indicated in the simulated field profile for an incident wavelength of 450 nm (Figure 4c), this absorption resonance is primarily due to a strong field enhancement localized at the Au–air interface near the top surface of the film.

**Figure 4:**
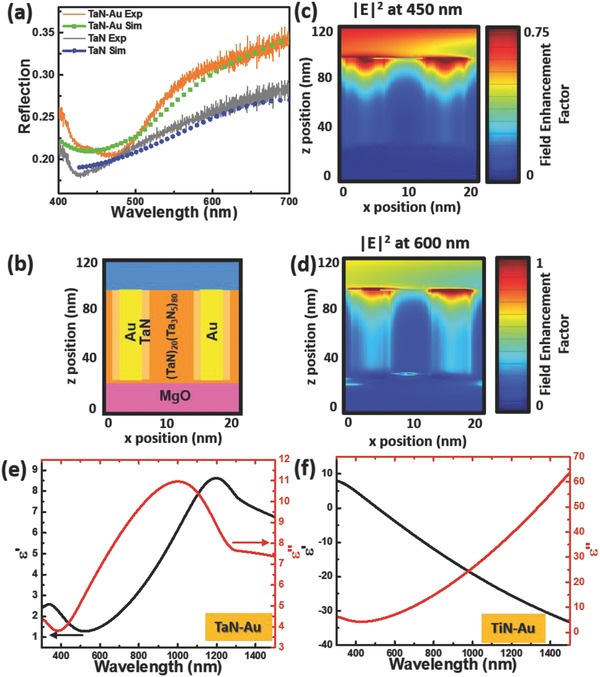
a) Comparison of the experimental and simulated reflections of the TaN with and without gold inclusion. c,d) The electric field enhancement at 450 and 600 nm, respectively, of b) the model geometry composed of gold pillars with a TaN cladding in a matrix of 20:80 TaN:Ta_3_N_5_ on MgO. Complex dielectric functions of e) TaN–Au and f) TiN–Au.

At longer wavelengths increased reflection has been observed compared with the control samples without Au nanopillars. Some of the increase in reflection is also due to scattering from the plasmon resonance of Au nanopillars at the air interface. In addition, in order to obtain a good match between the optical simulations and experimental reflectance spectra, it was also necessary to account for two distinct regions in the nitride matrix with slightly modified dielectric functions for the TaN. Consistent with the Raman analysis above, the best match with experiments was obtained when the region directly surrounding the Au nanopillar corresponds to stoichiometric TaN, and the rest of the matrix has a dielectric function characteristic of TaN with a slight increase in nitrogen concentration, labeled as 20:80 TaN:Ta_3_N_5_ in Figure 4. The dimensions of each region in the simulation are comparable to the regions of distinct contrast in the STEM HAADF image in Figure 2. The Ta:N composition estimation is also consistent with the STEM and EDS analysis results. In the simulated field profile for 600 nm incident light (Figure 4d) it is observed that a large fraction of the scattered radiation is also due to excitation of a longitudinal mode down the length of the nanopillar and the cladding around the nanopillar, similar to a coaxial waveguide mode. In addition, some backscattering is also due to the Au interface with the MgO support. Overall, it is clear that optical modes corresponding to individual pillar resonance, near 450 nm, and coupled resonances between the nanopillars and the dielectric matrix at longer wavelengths contribute to the overall optical properties of the APL.

Comparison of experimental and simulated reflection of TiN with or without Au is shown in Figure S9a (Supporting Information). Electrical field enhancement at 480 nm (Figure S9c, Supporting Information) and 600 nm (Figure S9d, Supporting Information) of TiN–Au has been observed based on the geometry shown in Figure S9b (Supporting Information). Similarly, Au nanopillar resonance coupled with Au nanopillar–TiN matrix resonance can be achieved. Experimentally measured reflectance of TaN on STO and ZrN on MgO, with or without Au is also presented in Figure S8c,d (Supporting Information), respectively. Transmittance and reflectance of TaN and TaN–Au in the infrared range were also measured and shown in Figure S10a,b (Supporting Information). Both transmittance and reflectance decrease after incorporating Au nanopillars, which indicates more light absorption by Au nanopillars in the infrared regime.

Furthermore, the complex optical dielectric functions of TaN–Au and TiN–Au on MgO were derived from spectroscopic ellipsometry measurements. As shown in Figure 4e,f, the complex dielectric constants of TaN–Au and TiN–Au were extracted from the experimental and fitted ellipsometric phi (ψ) values in Figure S11 (Supporting Information). The real part of the dielectric permittivity (*ε′*) of TaN–Au is positive throughout the entire measured wavelength range (300–1500 nm), while the real part of the permittivity for TiN–Au reaches negative for the wavelength higher than ≈500 nm, because of the metallic nature of both TiN and Au. The angular dependent and polarization‐resolved reflectivity was also measured and simulated to demonstrate the optical anisotropy of the nitride–Au films, as shown in Figures S12 and S13 (Supporting Information) for TaN–Au and TiN–Au, respectively. The results indicate the anisotropic optical response of the film, largely because of the anisotropic vertical pillar structure. The simulated results reproduced the relative angular dependent reflectivity and spectral trend of the experimental data well for both cases (see details in the Supporting Information).

To implement this new hybrid nitride–metal metamaterials for potential device fabrication and on irregular surfaces, desired mechanical properties such as a combination of high hardness (*H*) and excellent ductility are essential, especially for optoelectronic devices on flexible substrates and metamaterials coated on irregular surfaces. Transition metal nitrides are well‐known refractory materials with superior hardness and high temperature stability, which have been widely used as superhard and wear resistance coatings.^45^, ^46^ Typically, metals (Au in this case) are mechanically softer (e.g., *H*_Au_ ≈ 100–300 MPa) compared to nitrides (*H* ≈ 10–20 GPa). Thus, the mechanical properties of such hybrid nitride‐metal materials with APL are worth exploring. **Figure** **5**a shows a typical TiN–Au film deposited on an irregular glass surface (1 in. × 1 in.) with excellent uniformity and coating adhesion properties, which indicates large‐area conformal growth of the films with great potential of scale‐ups. To determine the hardness (*H*) and elastic moduli (*E*) of the hybrid nitride–metal films, nanoindentation with different loads and indentation depths was conducted. Figure 5b shows multiple load–displacement curves of TaN–Au sample, with the inset of a typical scanning probe microscopy (SPM) image of the film after nanoindentation. The maximum indentation depth was controlled below 15% of the total film thickness to avoid substrate effect. *H* and *E* measured at various locations are plotted in Figure 5c, with the average *H* and *E* calculated to be around 11.8 and 233.9 GPa, respectively. Very minor deviations of these data also indicate good film quality of TaN–Au. Interestingly, these values for the hybrid TaN–Au film are in the same range with reported values for pure TaN, suggesting that the overall hardness of the hybrid film is based on TaN.^47^ Another interesting observation is no crack formation in the SPM images after indentation indicating the ductile nature of the hybrid coatings which is ideal for coating on irregular surfaces. Similarly, the load–displacement curves of the TiN and TiN–Au films are shown in Figure 5d,e, respectively. Their calculated *H* and *E* are plotted and compared in Figure 5f. *E* of TiN–Au is 258.1 GPa, slightly higher than that of TiN film, 253.6 GPa; while the hybrid TiN–Au film exhibits a high *H* value of 23.3 GPa compared with the pure TiN film of 14.2 GPa. The enhanced mechanical properties in the hybrid case could be mainly attributed to the better nitrogen stoichiometry in the nitride matrix.^48^, ^49^, ^50^ It is also possible that the additional interface regions between the nitride matrix and Au nanopillars could have impact on the overall enhanced hardness, which has been realized in metal/nitride superlattices.^51^, ^52^ Au nanopillars can accommodate substantial plasticity and thus provide overall film ductility, while the hard nitride matrix can effectively block the dislocations motions via metal/nitride interface and thus maintain the overall high hardness of the hybrid films. Overall, the superior hardness and high ductility of the hybrid nitride–metal metamaterials combined with the unique optical properties all suggest their great potentials for tunable plasmonic coatings on large area and irregular surfaces.

**Figure 5:**
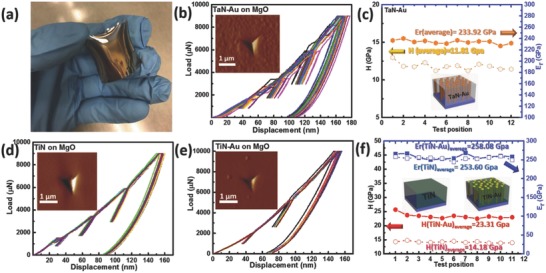
a) TiN–Au film deposited on a large‐scale curved glass. b) Multiple load–displacement curves of b) TaN–Au, d) TiN, and e) TiN–Au, with the inset of a typical SPM image of the film after nanoindentation. c) *H* and *E* of TaN–Au measured at various locations. f) Comparison of *H* and *E* of TiN and TiN–Au measured at various locations.

The novel nanostructured metamaterials and unique optical properties of the hybrid nitride–metal plasmonics nanostructures in VAN form are reported. The new platform with self‐assembled, highly epitaxial, large‐scale ordered Au nanopillars in nitride matrix presents exceptional optical and plasmonic response, signature of inversion symmetry breaking, excellent optical property tunability based on the nanopillar density and ordering, and large flexibility of materials combinations. Other plasmonic metals such as Ag, Cu, etc., could also be grown with transition metal nitrides to achieve novel performance. Combined with their large‐area one‐step coating capability on irregular surfaces and excellent mechanical performance (i.e., superior hardness, high ductility), the hybrid nitride–metal nanostructures could find enormous applications as tunable photonic structures for future optical devices and components, plasmonics structures for enhanced chemical‐ and biosensors and catalysis.

## Experimental Section

*Sample Fabrication*: Self‐assembled epitaxial TaN–Au, TiN–Au, and ZrN–Au vertically aligned nanocomposite thin films were deposited on single crystal MgO (001) and SrTiO_3_ (001) substrates, by a pulsed laser deposition system with a KrF excimer laser (Lambda Physik Compex Pro 205, λ = 248 nm, 5 Hz). Au foil pieces were sticked on nitride targets for the depositions. The laser beam was focused onto the target surface at an incident angle of 45° obtaining an energy density of about 3.0 J cm^−2^. Before deposition, the base pressure was pumped to lower than 1 × 10^−6^ Torr, and the films were deposited in vacuum condition with substrate temperature of 700 °C. After depositions, the samples were naturally cooled down to room temperature under high vacuum.

*XRD, TEM, and STEM HAADF Imaging*: The microstructure of the films was characterized by XRD (Panalytical X'Pert X‐ray diffractometer) and TEM (FEI Tecnai G2 F20 ST Materials). FEI Titan G2 80‐200 microscope with a Cs probe corrector and TEAM 0.5, a modified FEI Titan microscope with a special high‐brightness Schottky field emission electron source and an improved hexapole‐type illumination aberration corrector, were employed to record the STEM images in HAADF mode. The samples used for TEM and STEM analysis were prepared by a standard manual grinding and thinning procedure followed by final ion polishing in a precision ion polishing system (PIPS 691, Gatan).

*EDS Chemical Mapping*: A FEI Titan G2 80‐200 STEM with a Cs probe corrector and ChemiSTEM technology (XFEG and SuperX EDS with four windowless silicon driftdetectors) operated at 200 kV was used for EDS chemical mapping. EDS spectral images were acquired with an electron probe of size < 1.2 Å, convergence angle of 18.1 mrad, and current of ≈100 pA. Spectral images were acquired as a series of frames, where the same region was scanned multiple times. The frames were spatially drift‐corrected to build up spectral image data using a reference HAADF image. The instantaneous dwell time on each pixel was 20 µs, and a typical frame was 400 × 400 pixels. Spectral image collection typically took about 2000 s, yielding a total per pixel dwell time of about 12 ms.

*Optical and Raman Measurements*: Reflection spectra were measured with an optical microscope (Witec Alpha 300). Broadband, nonpolarized light‐emitting diode (LED) illumination at normal incidence was focused with an numerical aperture (NA) = 0.4 objective. Reflection spectra were normalized by comparison to an aluminum mirror. It was note that photoluminescence from the MgO substrate dominated the measured optical signal for wavelengths longer than ≈650 nm, and this spectral range was therefore omitted in the presented optical data. Raman spectra were obtained using the same microscope (Witec Alpha 300) in a confocal excitation and collection configuration. A 532 nm laser beam was focused on the surface of each using an NA = 0.9 objective. In the paper, the Raman scattering intensity was normalized to the peak intensity from each signal.

*SHG Measurement Setup*: SHG measurements were carried out using an amplified Ti:sapphire laser system, which produced pulses with a center wavelength of 780 nm (1.59 eV), a duration of ≈70 fs, and a repetition rate of 250 kHz. Using a 780 nm probe beam, SHG signal of 390 nm was generated. The SHG intensity was detected by a photomultiplier tube with lock‐in detection after filtering out the 780 nm beam. The polarization of the incident light was controlled by a half‐wave plate, and the SHG signal in the reflection from the sample with an angle of incidence at 45° was measured for either p (P‐out) or s (S‐out) polarizations.

## Conflict of Interest

The authors declare no conflict of interest.

## Supporting information

Supplementary
