# Supplementary material for: Nanoscale Artificial Plasmonic Lattice in Self‐Assembled Vertically Aligned Nitride–Metal Hybrid Metamaterials
Source: Adv Sci (Weinh). 2018 Apr 27;5(7):1800416. doi: 10.1002/advs.201800416 (PMC6051386; doi:10.1002/advs.201800416)
Supplement: Supplementary file 1 — Supplementary [file ADVS-5-1800416-s001.pdf]

Copyright WILEY-VCH Verlag GmbH & Co. KGaA, 69469 Weinheim, Germany, 2018.

Supporting Information

for *Adv. Sci.,* DOI: 10.1002/advs.201800416

Nanoscale Artificial Plasmonic Lattice in Self-Assembled Vertically Aligned Nitride–Metal Hybrid Metamaterials

*Jijie Huang, Xuejing Wang, Nicki L. Hogan, Shengxiang Wu, Ping Lu, Zhe Fan, Yaomin Dai, Beibei Zeng, Ryan Starko-Bowes, Jie Jian, Han Wang, Leigang Li, Rohit P. Prasankumar, Dmitry Yarotski, Matthew Sheldon, Hou-Tong Chen, Zubin Jacob, Xinghang Zhang, and Haiyan Wang**

Copyright WILEY-VCH Verlag GmbH & Co. KGaA, 69469 Weinheim, Germany, 2017.

Supporting Information

Nanoscale Artificial Plasmonic Lattice in Self-Assembled Vertically Aligned Nitride-Metal Hybrid Metamaterials

*Jijie Huang, Xuejing Wang, Nicki L. Hogan, Shengxiang Wu, Ping Lu, Zhe Fan, Yaomin Dai, Beibei Zeng, Ryan Starko-Bowes, Jie Jian, Han Wang, Leigang Li, Rohit Prasankumar, Dmitry Yarotski, Matthew Sheldon, Houtong Chen, Zubin Jacob, Xinghang Zhang and Haiyan Wang**

Optical Modeling: Full wave optical simulations were performed using a commercial finite-difference time-domain solver to calculate reflection spectra for 3-D geometries modeled from the sample morphological characterization. Wavelength dependent optical constants for gold,[1] TiN,[2] TaN,[3] and and non-stoichiometric Ta_3N_5[4] were taken from various literature sources. The refractive index of magnesium oxide was set to a constant value 1.745 based on its average value in the visible region.[5] Further details of the specific simulation geometry and modeling parameters are provided below.

Extended Simulation Description: The titanium nitride control film was based off of an 85 nm thick smooth TiN on a MgO substrate. For the sample with gold it was based off of gold pillars placed according to a top down TEM image placed in this same TiN film. For both perfectly matching layer boundary conditions were used on the top and bottom faces while the four edges along the matrix used periodic boundary conditions to simulate an infinite surface. A plane wave source was used to inject light with wavelengths between 400 and 800 nm with polarization along the x-axis. Reflection data was obtained using a power monitor placed above the material behind the source. A mesh of 0.4 nm in x and y and 1.2 in z directions were used to resolve the features.

The tantalum nitride control film was built with surface roughness to resemble an AFM image with a refractive index that was tuned between that of TaN and Ta_3N_5 to match experimental data. This simulation was done using PML boundary layers on all edges and a large simulation area to avoid introducing grating effects, though the same monitors and source were used. The tantalum nitride with gold was based off vertical pillars of gold in a 20:80 TaN:Ta_3N_5 matrix with root mean square surface roughness of 10 nm and thickness of 85 nm where the 3nm radius around each gold pillar was made up of TaN all of which was placed on a MgO substrate. Periodic boundary conditions, as well as the same source injection and monitors were used to record data. A mesh of 0.4 nm in x and y and 0.9 nm in z was used to resolve the gold pillars.

Infrared Transmission and Reflection Experiment Set-up: Infrared transmission measurements of the samples were taken with a Nicolet iS 50 FT-IR spectrometer. Measurement of signal passing through the samples was compared with background measurements where the signal was passed through the MgO substrate. The calculation for % Transmission is as follows:

$$\%T = \frac{I_{substrate+film}}{I_{substrate}}$$

Where $I_{substrate+film}$ is the intensity spectrum detected when passing the signal through the sample (MgO substrate + film of particular sample) and $I_{substrate}$ is the intensity spectrum detected when passing the signal through the MgO substrate alone.

The reflection spectra were measured using the standard FTIR microscope (HYPERION 2000). Infrared light is generated from a globar source and focused on the sample with a 15× IR objective (NA=0.4). The beam size was determined to be about 100μm×100μm by a knife edge aperture. The reflection light from the sample was refocused on a mercury cadmium telluride (MCT) detector. The frequency resolution was 2 cm^{-1} and every spectrum was averaged over 100 scans. The reflection spectrum of the sample was normalized to a gold mirror.

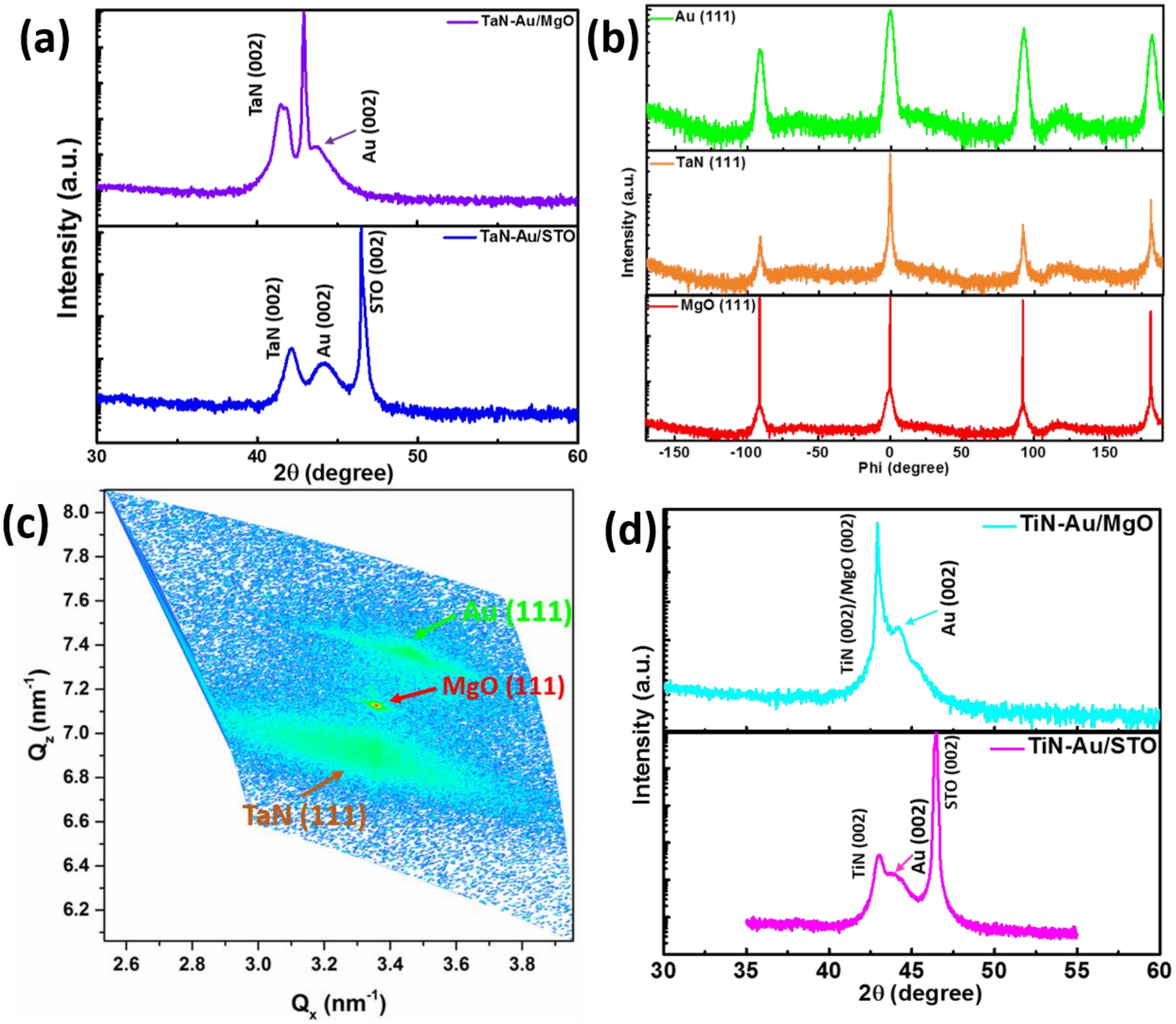

Figure S1. Phase identification of nitride-metal VAN thin films in this work. (a) θ-2θ XRD patters of TaN-Au thin films on MgO (001) and STO (001) substrates; (b) φ-scans of Au (111), TaN (111) and MgO (111) with 4-fold symmetry indicate the perfect in-plane matching of both TaN and Au; (c) Reciprocal space map (RSM) of TaN-Au on MgO to show the induced strain in the film; (d) θ-2θ XRD patters of TiN-Au thin films on MgO (001) and STO (001) substrates, to show the versatile selection of nitride matrices.

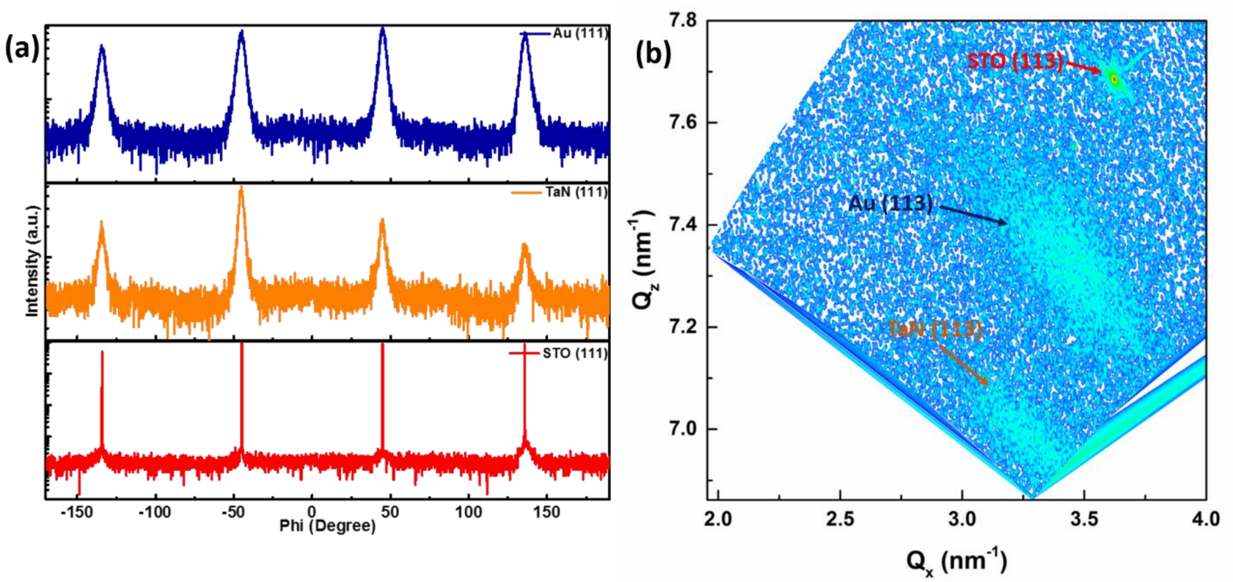

Figure S2. (a) φ-scans of Au (111), TaN (111) and STO (111) with 4-fold symmetry and (b) Reciprocal space map (RSM) of TaN-Au film on STO (001) substrate.

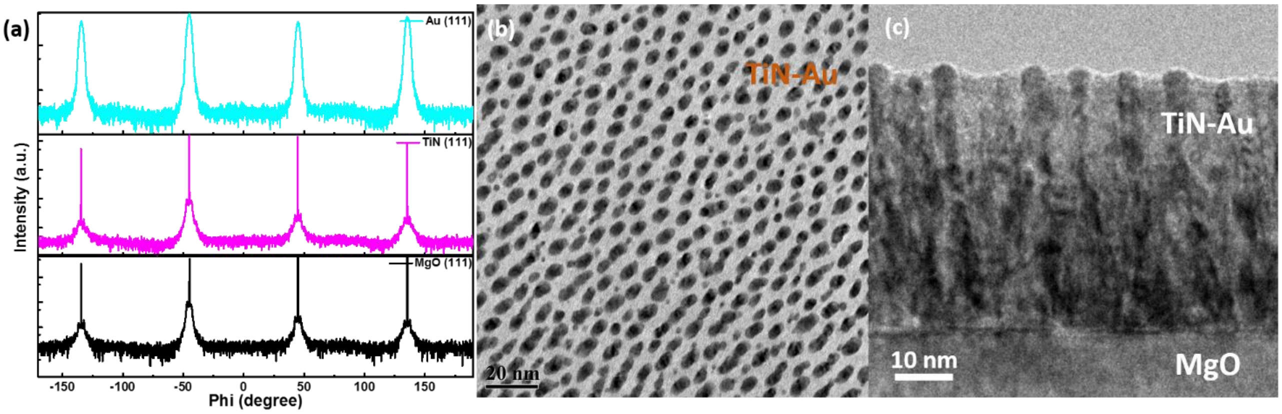

Figure S3. (a) φ-scans of Au (111), TiN (111) and MgO (111) with 4-fold symmetry of TiN-Au film on MgO (001) substrate; Low-magnification (b) plan-view and (c) cross-sectional TEM images of the film.

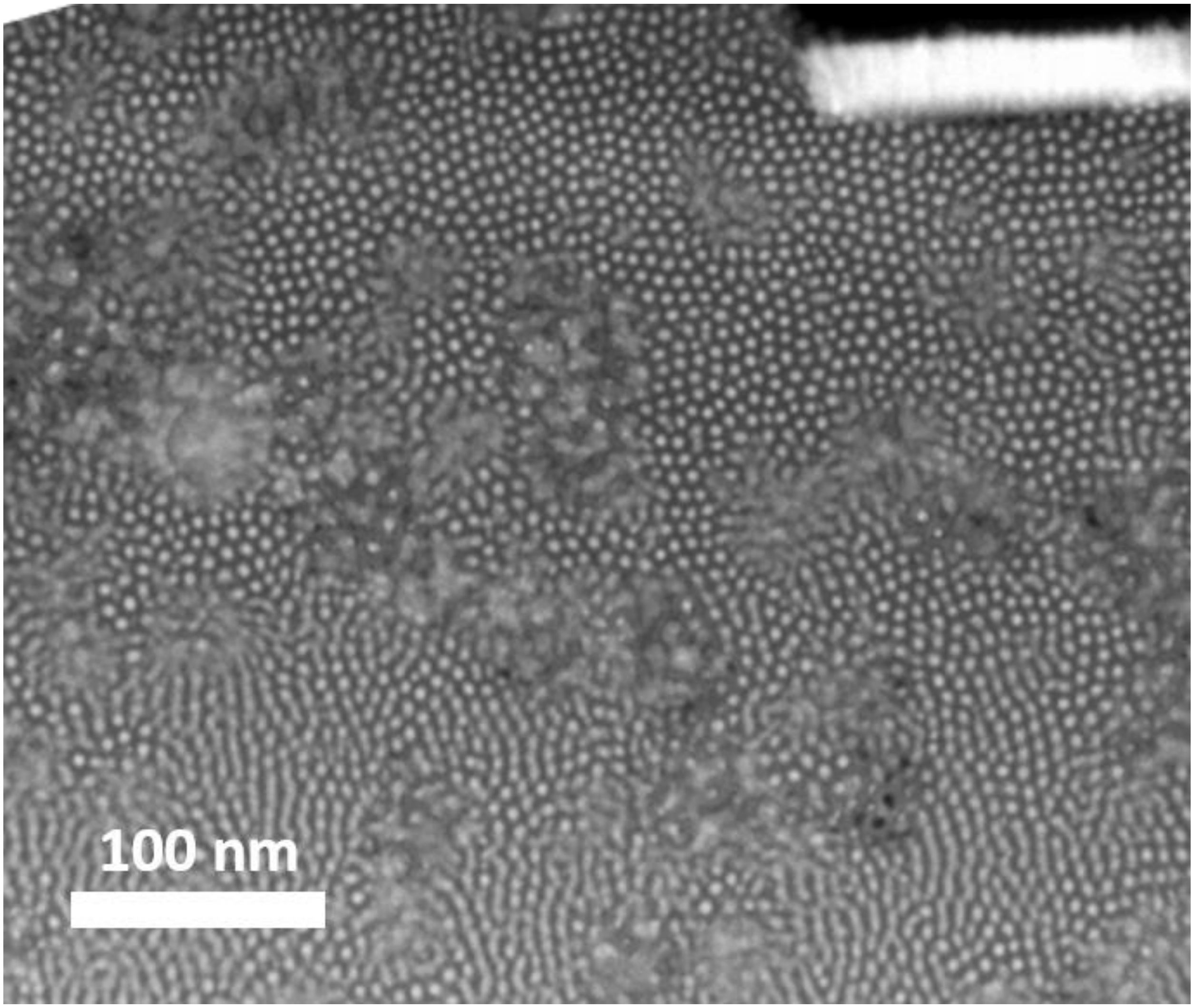

Figure S4. Low-mag STEM image of TaN-Au on MgO to show the large-scale uniformity of the film.

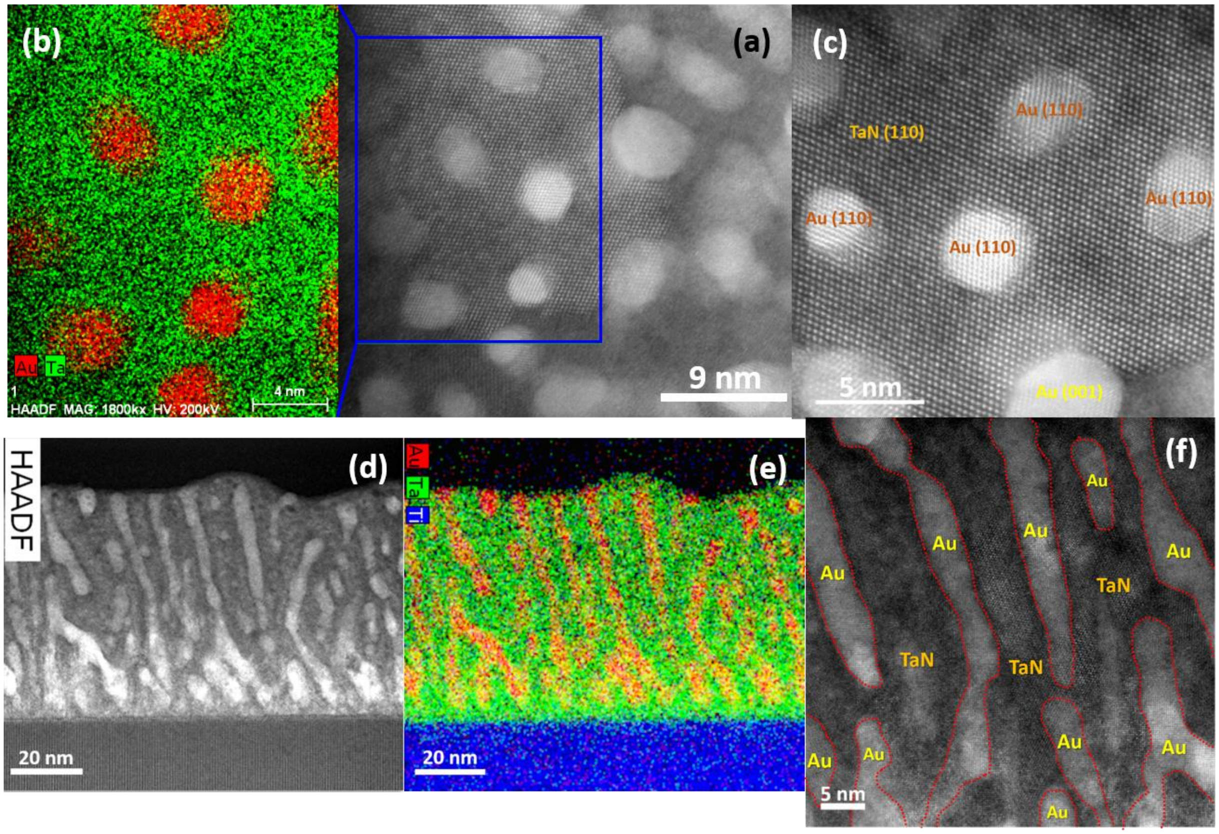

Figure S5. (a) Typically plan-view STEM image of the TaN-Au flim on STO (001) substrate with (b) corresponding EDS mapping of selected area; (c) High resolution plan-view STEM HAADF image of particular region shows TaN (110) and Au (110) phases; (d) Typically cross-sectional STEM image of the film with (e) corresponding EDS mapping of selected area; (f) Enlarged STEM image shows imperfect Au nanopillars in the film on STO (001) substrate.

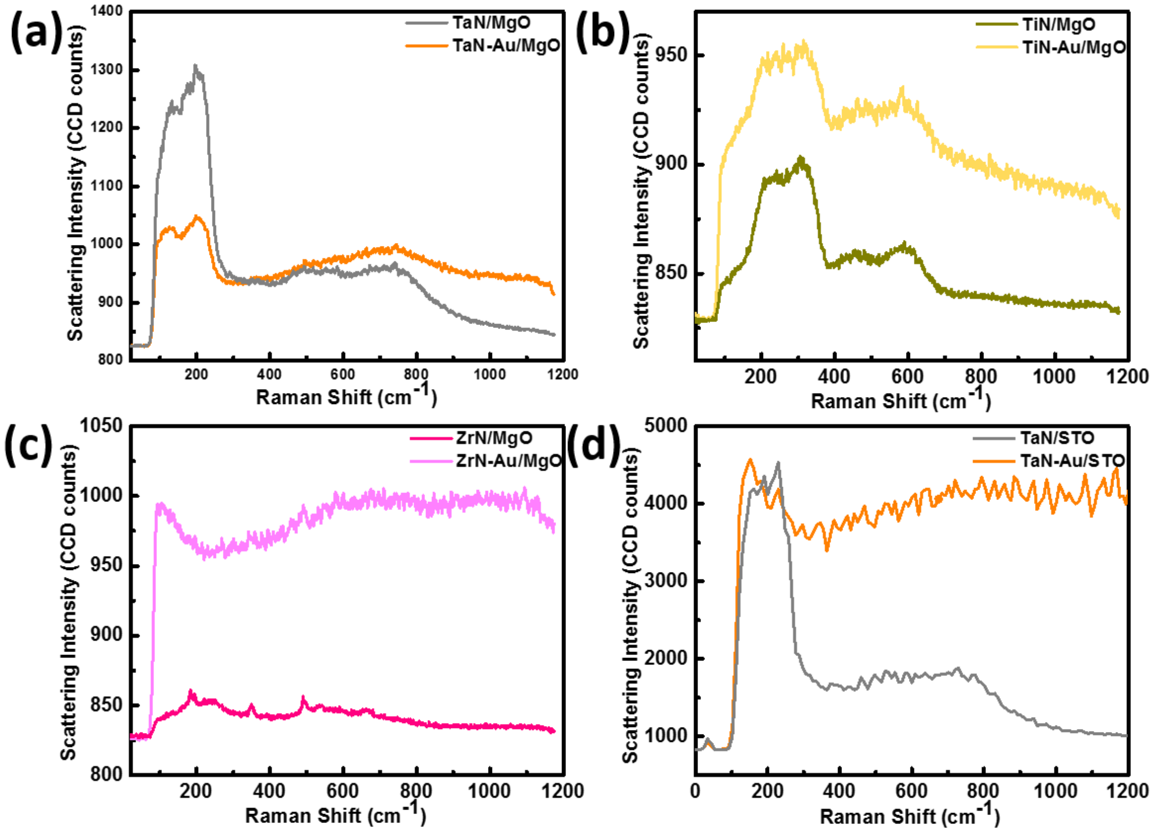

Figure S6. Raw Raman spectra of (a) TaN; (b) TiN; (c) ZrN thin film with and without gold on MgO substrate and (d) TaN thin film with and without gold on STO substrate.

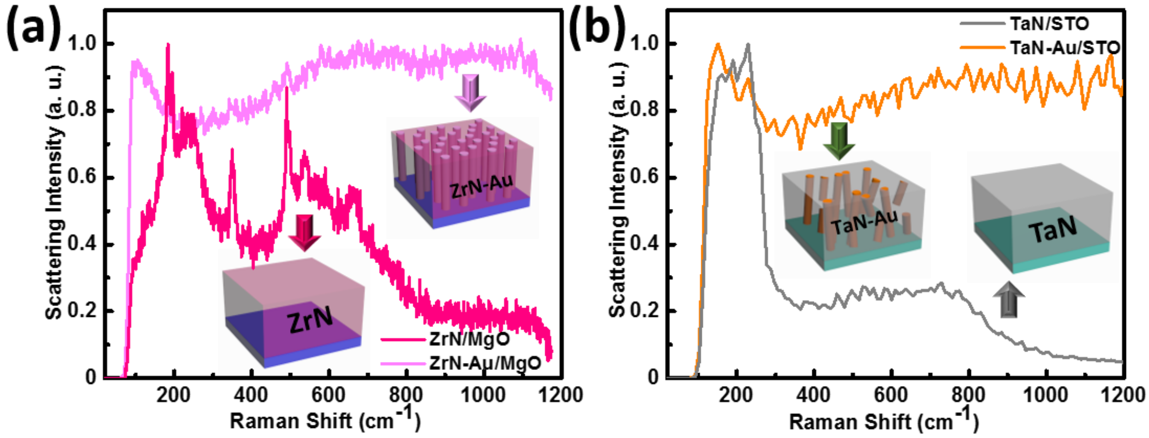

Figure S7. Normalized Raman spectra of (a) ZrN and ZrN-Au on MgO, and (b) TaN and TaN-Au on STO; The insets are their corresponding schematic illustrations.

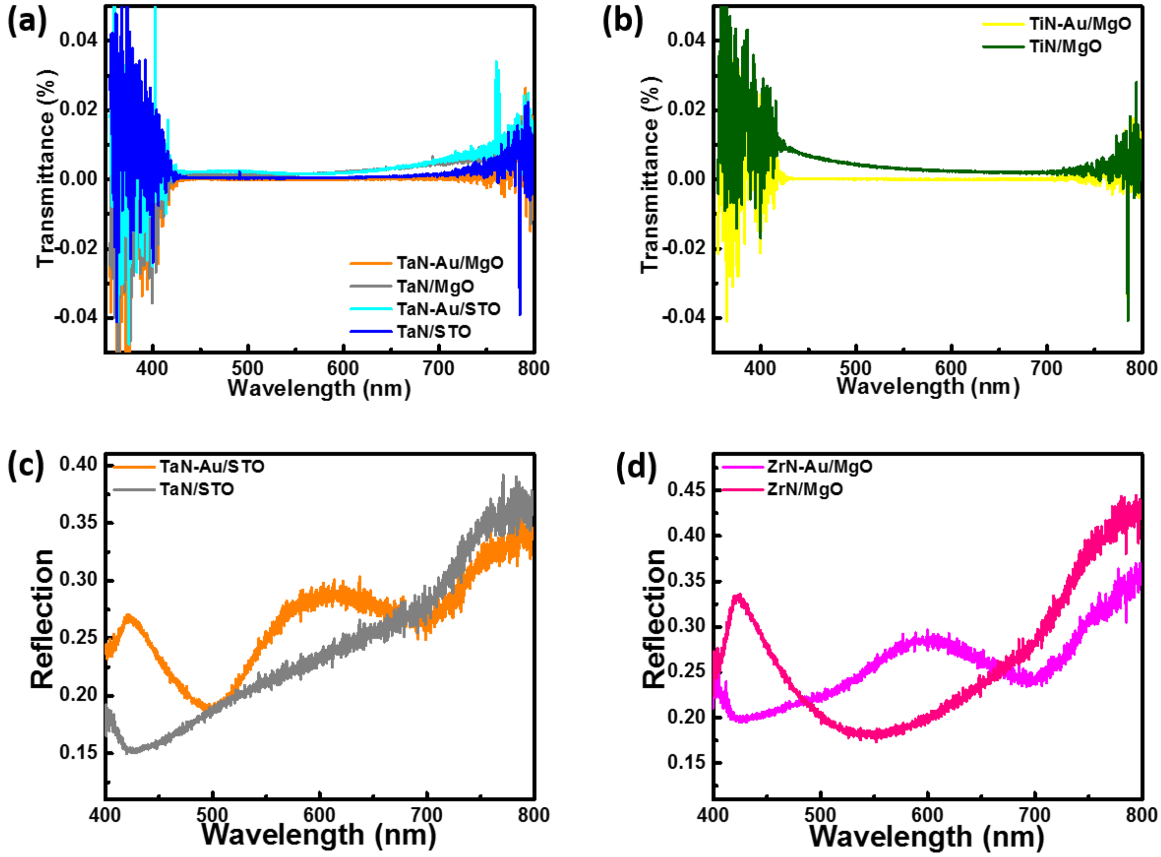

Figure S8. Visible range transmittance of (a) TaN with or without Au on MgO and STO; (b) TiN with or without Au on STO; Visible range reflectance of (c) TaN with or without Au on STO; (d) ZrN with or without Au on MgO.

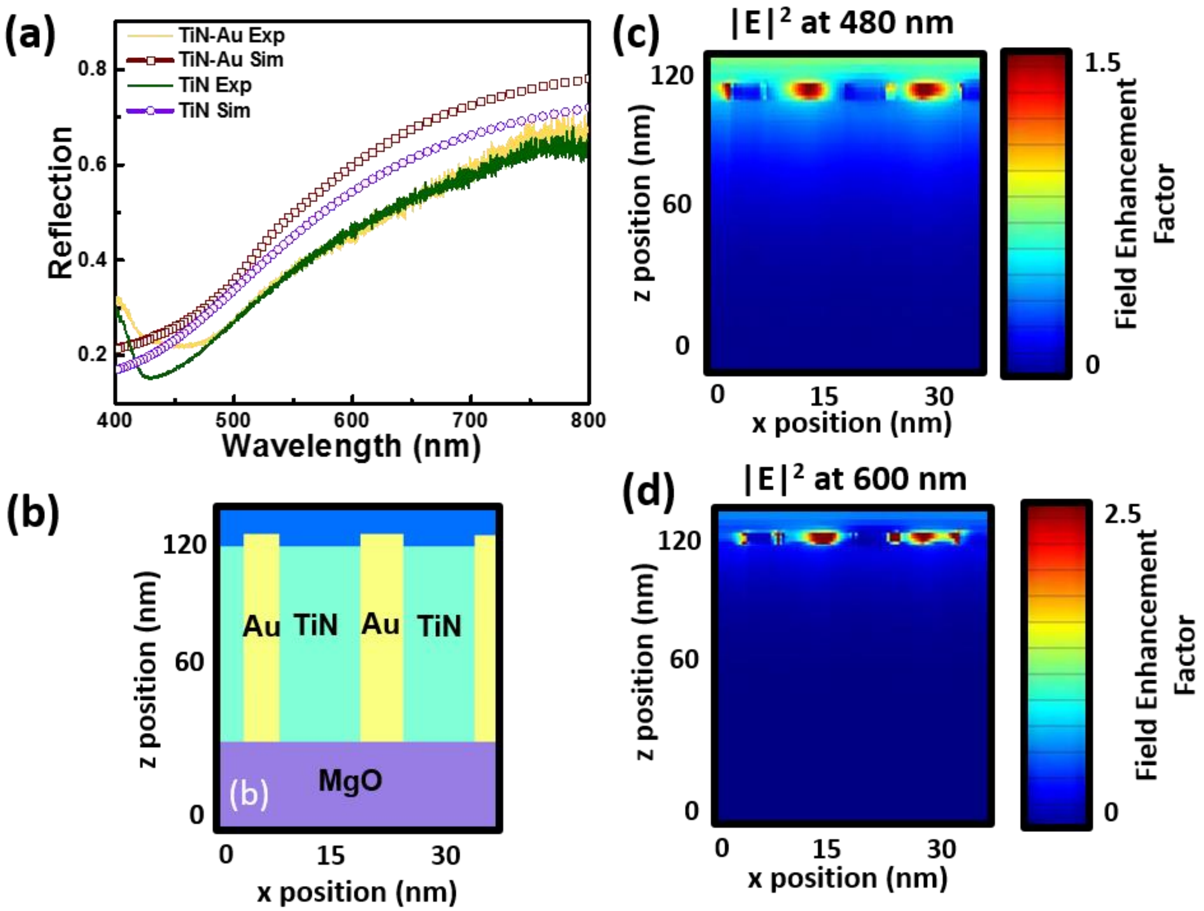

Figure S9. (a) Comparison of the experimental and simulated reflections of the TiN with and without gold inclusions. (c) and (d) are the electric field enhancement at 480 and 600 nm of the model geometry respectively in (b) composed of gold pillars with TiN matrix on MgO.

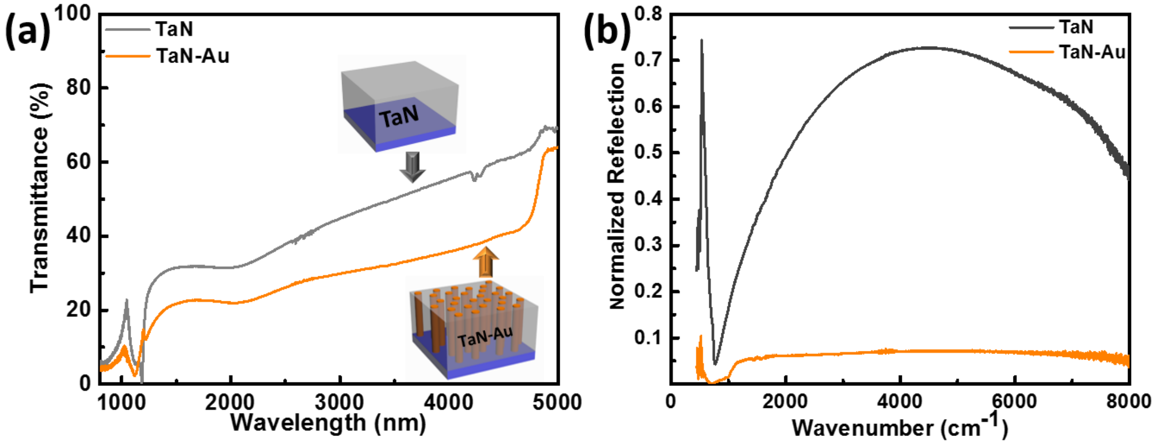

Figure S10. (a) Transmittance and (b) reflectance in infrared range of TaN with or without Au on MgO.

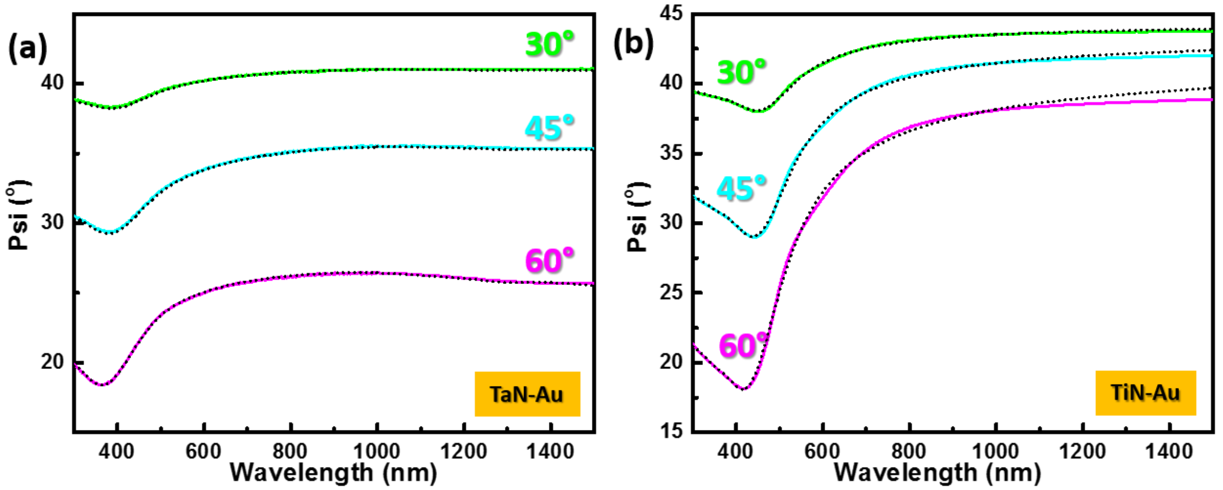

Figure S11. Experimental (solid points) and fitted (solid lines) components at different angles of the ellipsometric parameter (ψ) vs. wavelength of (a) TaN-Au and (b) TiN-Au on MgO substrate.

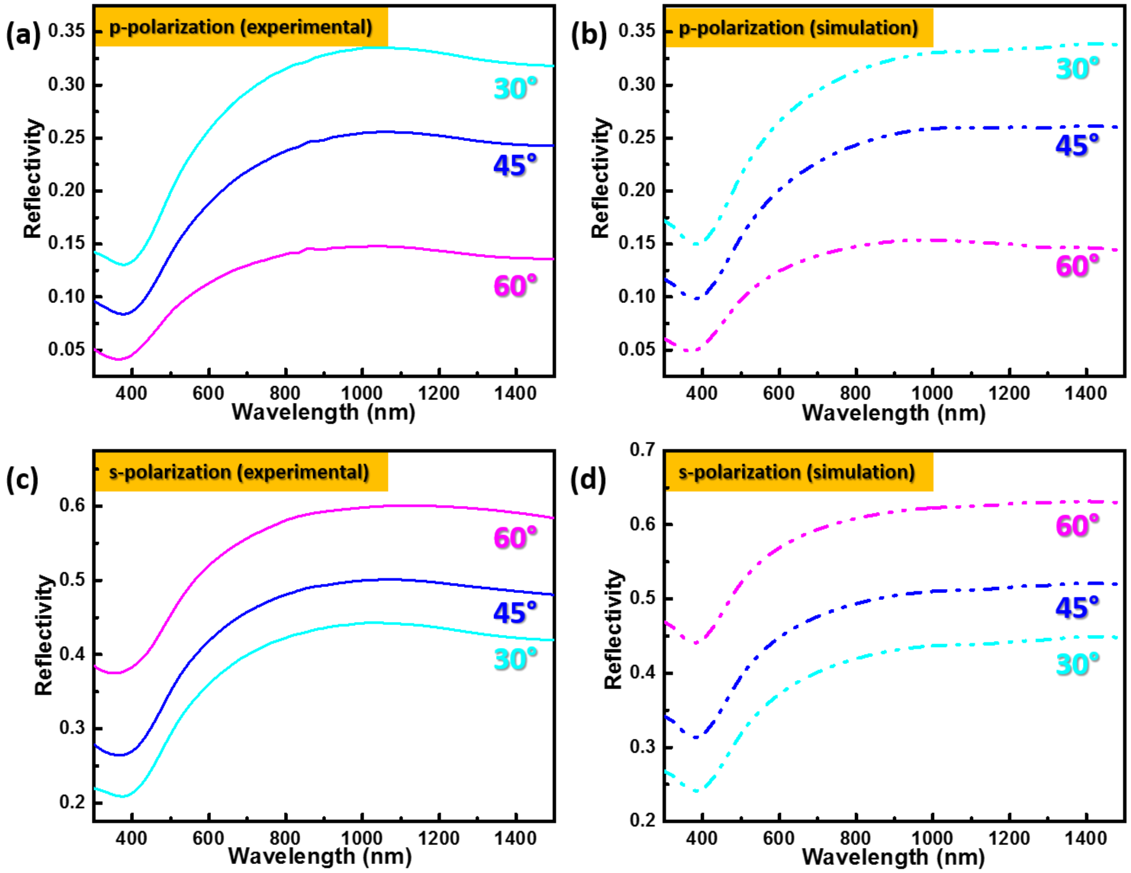

Figure S12. Reflectivity spectra of the TaN-Au film for different angles of incidence for (a) p-polarized and (c) s-polarized incident lights; Simulated reflectivity spectra for (b) p-polarized and (d) s-polarized incident light.

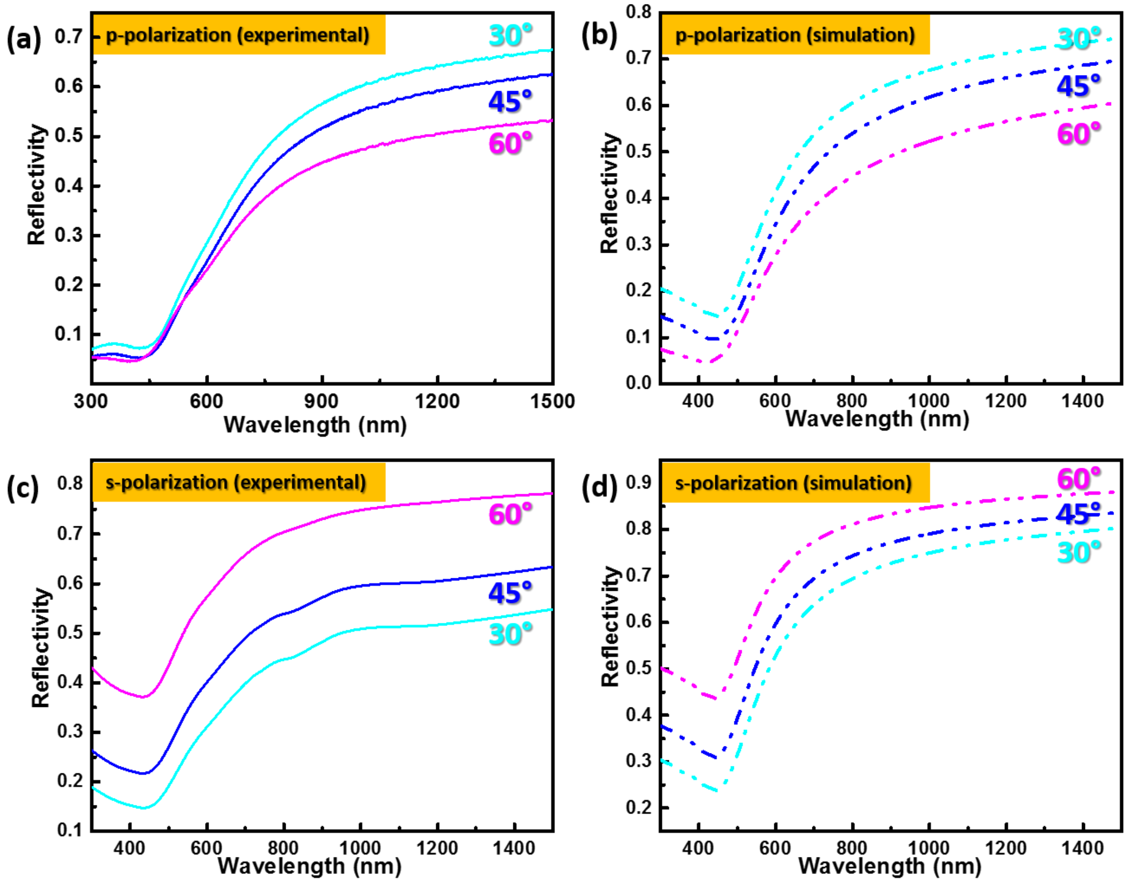

Figure S13. Reflectivity spectra of the TiN-Au film for different angles of incidence for (a) p-polarized and (c) s-polarized incident lights; Simulated reflectivity spectra for (b) p-polarized and (d) s-polarized incident light.

Variable angle ellipsometry experiments were carried on a RC2 spectroscopic ellipsometer (J.A. Woollam Company). Two parameters Psi (ψ) and Delta (Δ) were obtained by the ellipsometry measurements, which are related to the ratio of the reflection coefficients for the light of p-polarization r_p and s-polarization r_s: $r_p/r_s = \tan(\psi)\exp(i\,\Delta)$. Then, the complex dielectric constants were obtained by fitting the ellipsometry data using different models in the VASE software. Here, TaN-Au sample was fitted by general oscillator layer (GOL) model with three Tauc-Lorentz oscillators, while TiN-Au was fitted by general oscillator layer (GOL) model with one Drude and

two Tauc-Lorentz oscillators. All fitting resulted in a reasonably small mean squared error (MSE) of less than 5.

References:

[1] Johnson, R. & Christy, R.W. Optical Constants of the Noble Metals. *Phys Rev. B.* **6 (12)**, 4370-4379 (1972).
[2] Palik, E. Handbook of Optical Constants of Solids I-III. (Academic Press: New York, NY, USA, 1998).
[3] Aouadi, S.M. & Debessai, M. Optical properties of tantalum nitride films fabricated using reactive unbalanced magnetron sputtering. *J. Vac. Sci. Technol. A.* **22 (5)**, 1975-1979 (2004).
[4] Langereis, E., Heil, S. B. S. Knoops, H. C. M., Keuning, W., van de Sanden, M. C. M. & Kessels, W. M. M. In situ spectroscopic ellipsometry as a versatile tool for studying atomic layer deposition. *J. Phys. D: Appl. Phys.* **42**, 073001 (2009).
[5] Stephens, R. & Malitson, I. Index of refraction of magnesium oxide. *Journal of Research of the National Bureau of Standards.* **4 (49)**, 249 (1952).
